# One decade of “English as a medium of instruction” (EMI) in healthcare education

**DOI:** 10.3389/fmed.2024.1296563

**Published:** 2024-02-29

**Authors:** Munassir Alhamami

**Affiliations:** English Department, Faculty of Languages and Translation, King Khalid University, Abha, Saudi Arabia

**Keywords:** healthcare education, English as a medium of instruction (EMI), medical education, language of instruction, higher education

## Abstract

**Introduction:**

This paper analyzes published healthcare studies about “English as a medium of instruction” (EMI), indexed in the Scopus database from 2013 to 2022.

**Methods:**

The author used published criteria of systematic reviews and limited the findings to healthcare education using several key terms; this returned 137 articles. The author then downloaded and carefully read the articles. The majority of articles (102) were deleted because they did not meet the selection criteria discussed in the methods section, thus the final list comprised 35 research studies. Next, the author analyzed the articles’ bibliometric indexes, such as author, funding information, context, research instruments, years of publication, place of publication, and citations. In addition, the key findings and recommendations of these studies were presented.

**Results and discussion:**

Most of the studies assessed were conducted in the last five years in Arabic speaking countries by non-language specialists, and the language of instruction was not the main focus of the studies. The studies were most often about attitudes of students, and used quantitative methods such as questionnaires. The results show diverse and conflicted results such as positive impacts and positive attitudes in some cases, negative impacts and attitudes in others, and preferences for either monolingual or bilingual approaches. The findings demonstrate the need for experimental and rigorous mixed methods studies that involve different stakeholders and are conducted by both applied linguists and healthcare education specialists. Future research should move beyond student attitudes and utilize rigorous mixed methods involving researchers from both linguistics and healthcare education to deepen our understanding of EMI’s complex impact in diverse contexts.

## Introduction

The English as a medium of instruction (EMI) policy has dominated healthcare education because English is the lingua franca of science (1). Macaro (2) defines EMI as “the use of the English language to teach academic subjects other than English itself in countries or jurisdictions where the first language of the majority of the population is not English” (p. 19). We refer to this definition in the selection criteria presented later in this study. The impact of EMI policy is a concern for researchers and policymakers in healthcare departments. The literature shows a diversity of conflicting results, as we will explore later in this study. There is therefore a need to conduct a systematic review in order to understand the outcomes of EMI research in healthcare colleges. The need to conduct more EMI research in order to determine patterns and trends has been emphasized by several researchers and international organizations (3). Many developing countries face policy challenges in deciding the language of instruction (4). There is a strong need to examine the influence of EMI in healthcare education.

The field of education research has firmly recognized EMI across both higher and secondary education levels (5). Researchers [e.g., (6)] stressed the need for further research in EMI, include the impact of EMI on content learning, specifically objective student outcomes, which remains understudied (7–9). There’s a need for high-quality research to inform stakeholders to what extent EMI affects learning through a second language (2). Macaro and Rose (6) highlight the need for research that examine the effect of EMI on student English proficiency, especially the type of linguistic knowledge that improves, balancing this against any potential negative impact on content learning. Research should clarify the specific language skills enhanced by EMI compared to general English learning (6). Additionally, there’s a need to understand the strategies EMI students use to navigate the challenges of tertiary education (10–12). Researchers [e.g., (6)] encourage contributions from underrepresented countries in EMI research.

The main objective of this study was to analyze the findings of research on EMI policy in healthcare education through a systematic review of the literature from the past decade. As far as we are aware, such a comprehensive review has not yet been conducted, and it will offer fresh insights to the body of existing literature. It will delineate the principal findings, chart the progression of EMI research themes, and forecast directions for subsequent scholarly endeavors, thereby informing policy development in healthcare education. This study will serve as an extensive resource for researchers, policymakers, and stakeholders vested in the integration of EMI within healthcare education. It will also provide international educationists with critical perspectives on language policy in the context of healthcare learning environments. The study will also propose areas necessitating further investigation, like the influence of EMI on the academic outcomes of healthcare students. By suggesting new research pathways, it will contribute to a more thorough exploration of EMI’s broader educational impact. Also, the study will project recommendations for healthcare education policymakers.

## Literature review

English as a medium of instruction in healthcare education has been a focus of diverse research, addressing its implementation, challenges, and outcomes. This literature review categorizes the extensive body of research into seven major themes, providing a comprehensive understanding of EMI’s complexities, diverse perspectives, and impacts across various contexts.

The studies in first theme focus on the perceptions, attitudes, and perspectives of students and instructors in different countries such as Saudi Arabia [e.g., (13, 14)], Korea [e.g., (15)], South Africa, [e.g., (16, 17)]; and Hong Kong (18). Research across various regions has assessed stakeholders’ perceptions of EMI in healthcare education. Alrajhi et al. (13) and Horwood et al. (19) found favorable views of EMI, noting its role in global connectivity and career development. In contrast, Al-Zubi et al. (20) and Saha et al. (21) identified a preference for native language instruction due to EMI’s linguistic challenges and resource scarcity. Alfakhry et al. (22) explored attitudes toward language translation in educational content, while Al-Zubi et al. (20) assessed the reception of Arabicized medical terminology. Dube and Mlotshwa (23) provided insights into nursing students’ perceptions in South Africa, adding depth to the discourse on EMI’s impact on learning environments. Negative perceptions of EMI have been shown to affect student behavior and outcomes, with peer influence also shaping engagement (24).

The second thematic cluster of studies within healthcare education centers on the multifaceted challenges posed by EMI. Al Zumor (25) explored the specific hurdles faced in scientific disciplines in Saudi Arabia, shedding light on student perceptions related to understanding lectures, communication, and pedagogical efficacy. Echoing this, Pomat et al. (26) explored the complex needs and obstacles encountered by nursing students and educators in Thailand, where instruction occurs in English, Thai, or a combination of both. Yang et al. (27) further expanded this discourse by examining the adaptive strategies employed by teachers and students within a Chinese EMI medical education context to overcome similar issues. Although Al Zumor (25) and Pomat et al. (26) emphasize the considerable challenges, such as student anxiety and insufficient teaching resources, Yang et al. (27) provide a counterbalance, suggesting that strategic adaptations and resource enhancement can effectively address these concerns.

The third theme in EMI healthcare education studies emphasizes textual and policy analysis. Alhamami and Almelhi (28) assessed the EMI policy’s effectiveness by evaluating Saudi Arabian healthcare college alumni’s academic records and experiences. Law et al. (29) scrutinized the mutual recognition arrangements among ASEAN nations, relating them to the professional mobility of health personnel in Cambodia. Additionally, Alsuliman et al. (30) scrutinized bilingual medical texts for Arabic-speaking students, assessing their educational efficacy. Addressing the adaptation of medical terminology, Lazer-Pankiv and Pysmenna (31) investigated how Latin terms are phonetically and orthographically adapted for EMI, analyzing the impact on foreign medical students’ terminological competence. Otomo (32) focused on Japanese healthcare licensure applicants, exploring how language training policies affect their career prospects in Japan. Mayberry (33) tracked the trajectory of Chinese medical graduates studying in English, highlighting implications for the UK medical field. They doing an English parallel course through a Freedom of Information search of the current UK medical register. These studies suggest that English proficiency is required for academic success (28, 29), yet Lazer-Pankiv and Pysmenna (31) and Otomo (32) caution against the complexities of EMI policy implementation in contexts where English is not the dominant language.

The fourth thematic area in EMI healthcare education research addresses pedagogy and curriculum support. Hijji (34) critically assessed the design of multiple-choice questions in nursing exams at Middle Eastern universities, applying a set of 22 principles to gauge their effectiveness. Kumar et al. (35) surveyed preferences for teaching methods among North Indian dental and medical faculties and students, aiming to optimize lecture strategies and educational tools. In the field of pharmacy education, Khan (36) examined the integration of technology as a pedagogical tool, assessing its impact on learning outcomes. Together, these studies underscore a demand for enhanced teaching approaches within EMI settings, with Hijji (34) identifying gaps in test construction, and Khan (36) advocating for technological advancements. Kumar et al. (35) complement these findings by advocating for teaching aids tailored to the specific needs of the local educational context.

The fifth theme in EMI healthcare education research focuses on the impact of student diversity and linguistic backgrounds. Mustonen and Strömmer (37) explored the growing presence of migrant students in Finland’s vocational education and their unique linguistic assets, suggesting a need for further studies on leveraging their multilingualism through translanguaging practices. Roshini et al. (38) examined the perceptions of dental students taught in multilingual settings, noting that non-English backgrounds could affect academic performance and self-assessment, with perceptions evolving throughout their studies. Ndawo (39) provided insights into nurse educators’ experiences with EMI, revealing a generally positive stance despite challenges like the shortage of skilled EMI instructors. These studies collectively reveal the influence of linguistic backgrounds on EMI adaptability and propose a more inclusive educational approach that values students’ language skills (37, 38).

The sixth thematic strand within EMI healthcare education research investigates stakeholder experiences. Møller (40) provided an account of Inuit nursing students grappling with language and cultural hurdles within a healthcare system influenced by Euro-Canadian and Danish norms. The study found that the Inuit students faced a number of challenges, such as language barriers, cultural differences, and a lack of support from their families and communities. Waterval et al. (41) explored the dynamics of international medical curriculum partnerships, noting the potential risks such as subpar curriculum execution and insufficient preparation for clinical practice in host nations. They underscore the necessity for further exploration into students’ perspectives on these transnational educational experiences. Salamonson et al. (42) examined the implications of a globalized student body in nursing programs, emphasizing the success tied to early language assistance. Together, these studies illustrate the complex interplay between EMI, cultural identity, and academic achievement, suggesting that while EMI presents certain challenges like cultural discord and language obstacles, it also offers significant benefits for educational development and global research competencies.

The final thematic focus lies on the interplay between students’ language proficiency and their academic understanding. Tenney et al. (43) established a significant link between English proficiency and overall academic achievement in a Hong Kong pharmacy program, surpassing the influence of scores in other subjects such as mathematics, chemistry, or Chinese. Schoepp (44) reinforced this by demonstrating how proficiency test results, like IELTS and TOEFL scores, align with GPA indicators, providing a predictive measure of academic success. Mann et al. (45) explored this further by linking verbal working memory, a key component for academic performance, with English language proficiency. Their findings suggest that even students with high English proficiency may encounter academic difficulties if they are non-native speakers, pointing to nuanced challenges in language acquisition that extend beyond test scores. These studies collectively underscore the critical role of language proficiency in EMI contexts, while also acknowledging the nuanced academic hurdles faced by non-native speakers (43–45).

The literature review reveals a nuanced landscape shaped by varied perceptions, systemic challenges, and the interplay of language proficiency with academic achievement. The favorable views of EMI, noted by Alrajhi et al. (13) and Horwood et al. (19), reflect its potential in globalizing healthcare education and broadening career prospects. However, the preference for native language instruction, as found by Al-Zubi et al. (20) and Saha et al. (21), indicates the need for a balanced approach that considers linguistic barriers and resource limitations. Challenges highlighted by Al Zumor (25) and Pomat et al. (26) emphasize the anxiety and resource inadequacies faced by students and faculty, while Yang et al. (27) propose adaptive strategies as a remedy. Policy and textual analyses by Alhamami and Almelhi (28) and Law et al. (29) support the benefits of EMI when English proficiency is robust, yet they also caution against the complexities involved in non-English dominant regions, as discussed by Lazer-Pankiv and Pysmenna (31) and Otomo (32).

The necessity for improved pedagogy is clear from the critiques of Hijji (34) and the technological integration suggested by Khan (36). The diverse backgrounds of students, explored by Mustonen and Strömmer (37) and Roshini et al. (38), call for an inclusive, multilingual approach to education, recognizing and utilizing the linguistic assets of students. Stakeholder experiences, particularly of groups like the Inuit studied by Møller (40) and international students in curriculum partnerships examined by Waterval et al. (41), reveal cultural and linguistic hurdles, yet also highlight the transformative potential of EMI in fostering global competencies. Finally, the critical role of language proficiency in academic success, as seen in the findings of Tenney et al. (43) and Schoepp (44), reinforces the need for language support in EMI programs. While EMI holds the promise of enhancing healthcare education by offering a global perspective, this review underscores the importance of addressing the linguistic, cultural, and pedagogical challenges that accompany its implementation. Ensuring students’ linguistic capabilities, fostering an inclusive environment, and providing effective teaching strategies are paramount for harnessing the full potential of EMI in healthcare education.

## Methodology

We used the Scopus database (accessed on 9 January 2023), a widely utilized and reliable source of data for literature reviews (46). PubMed and Medline (Ovid) content are a subset of Scopus. PubMed indexes around 6,000 journals, Scopus indexes around an additional 17,000 (total around 24,000) journals including most, but not all, of the content of the Embase database (47). Vitta and Al-Hoorie (48) reported that faculty members in Asia could be rewarded approximately three times more for publishing in a Scopus-indexed journal than in a locally indexed journal. Scopus is considered one of the main indexes of prestige within academia (49). Researchers have recommended the use of Scopus to analyze data because Scopus is broader than the other databases, and many good papers are indexed there (50).

**FIGURE 1:**
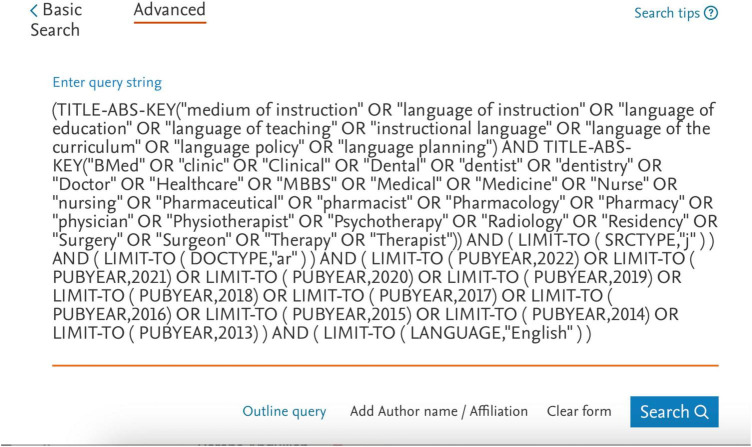
Researched keywords.

To conduct a systematic review of previous research, we established a set of inclusion and exclusion criteria following the review protocols developed by Macaro et al. (51) in their systematic review and their definition of EMI. First, we included all articles available within the database in the “final” or “in press” publication stage from 1 January 2013 to 31 December 2022. We used particular keywords that appeared in the article title, abstract, and keywords, adapted from Macaro et al. (51): “medium of instruction” OR “language of instruction” OR “English Medium of Instruction” OR “English as a Medium of Instruction” OR “Content and Language Integrated Learning.” In healthcare education, we adapted the following keywords from systematic reviews on healthcare education [e.g., (52–54)]: (“language policy” OR “language planning”) AND TITLE-ABS-KEY (“BMed” OR “clinic” OR “Clinical” OR “Dental” OR “dentist” OR “dentistry” OR “Doctor” OR “Healthcare” OR “MBBS” OR “Medical” OR “Medicine” OR “Nurse” OR “nursing” OR “Pharmaceutical” OR “pharmacist” OR “Pharmacology” OR “Pharmacy” OR “physician” OR “Physiotherapist” OR “Psychotherapy” OR “Radiology” OR “Residency” OR “Surgery” OR “Surgeon” OR “Therapy” OR “Therapist”). We limited the results to the last decade (the last ten years), 2013–2022, and to journal articles that were published in the English language (see Figure 1).

The results yielded 137 documents. Next, we exported the data to an Excel spreadsheet. The spreadsheet contained the following information: Authors, Author full names, Author IDs, Articles titles, Year of publication, Journal name, Article DOI, Article cited by, Article link, Authors, Affiliations, Article, Abstract, Indexed keywords, Author keywords, Funding details, Funding texts, Article references, Correspondence address, ISSN, Language of original document, Abbreviated source title, Document type, Publication stage, and Open access.

Next, the author downloaded the full manuscripts of these 137 articles and read the titles and abstracts of each paper to verify the relevance of the document to the scope of the study. If the main focus of the abstract did not explicitly reflect the language of the instruction, full articles were consulted to verify the relevance of the document to the study objectives. The following inclusion and exclusion criteria were adopted to narrow the selection of studies: (1) took place in instructional settings where the majority of the population was healthcare students such as nursing; (2) focused on contexts in which the participants spoke the instructional language as a foreign language; (3) recruited participants currently studying medical subjects, not English proficiency courses; (4) took place in higher education. Students in high school and secondary education contexts were excluded; (5) adopted empirical methods of data collection (e.g., interviews, questionnaires, observations, tests); and (6) one of the main objectives of the study or its findings involved discussion of the language of instruction. We also excluded conference proceedings, book reviews, Master’s dissertations, and Ph.D. theses, which meant that only peer-reviewed journal articles were included in the review.

The selected articles (*n* = 35) were then analyzed with the above information having been downloaded automatically from the database, the researchers having added other columns to insert information after reading each article carefully: location of the study, educational level, participant description, focus of the study, research methods and instruments, and key findings (see online Supplementary materials, for full list of the articles).

Here is a summarized description of the systematic review process. This summary provides an overview of the systematic review methodology, highlighting the main points from identification to the analysis of selected articles. For a full list of included articles and detailed information, refer to the online Supplementary materials. Table 1 illustrates the procedures and inclusion and exclusion criteria.

**TABLE 1:** Systematic review procedures summary.

1. Database and time frame:	Searched the Scopus database on 9 January 2023.
	Articles from 1 January 2013, to 31 December 2022, considered.
2. Criteria for consideration:	Included empirical studies in the final or in-press stage.
	Excluded conference proceedings, book reviews, dissertations, and theses.
	Limited to articles published in English.
3. Keyword search:	For the EMI field, terms from Macaro et al. (51):
	For healthcare education, keywords from systematic reviews [e.g., (52–54)]
4. Initial results and data management:	Obtained 137 documents initially.
	Exported details to an Excel spreadsheet, including bibliometric information and article specifics.
5. Screening and eligibility:	Downloaded and reviewed the full manuscripts based on titles and abstracts.
	Applied inclusion and exclusion criteria focusing on: • Instructional settings with healthcare students. • Contexts where the instructional language is a foreign language. • Participants studying medical subjects, excluding English courses. • Studies conducted in higher education settings. • Empirical data collection methods. • Studies with a primary focus on the language of instruction.
6. Selection of studies:	After the screening, 102 articles were excluded.
	35 articles met all criteria and were included for in-depth analysis.
7. Further analysis and documentation:	For the selected 35 articles, additional information such as the study location, educational level, participant description, study focus, research methods, and key findings was documented.

While the current study employs a rigorous methodology that effectively reaches its defined objectives, its scope necessitates acknowledging certain limitations that future research could address to further enrich our understanding of the field. First, focusing solely on English-language publications restricts the data’s richness and diversity, particularly in the context of language instruction. Significant research often emerges in the local languages of the study setting, potentially offering valuable insights. Future studies could significantly benefit from expanding the language scope to capture this valuable knowledge. Also, limiting the analysis to one decade offers a valuable snapshot, but a broader historical perspective could reveal fascinating trends and transformations in language instruction practices and theories. Extending the timeframe of future studies to include earlier research would illuminate the evolution of this dynamic field. In addition, peer-reviewed journals form the cornerstone of academic exploration, but valuable contributions also appear in conference proceedings, dissertations, and theses. These sources may harbor innovative ideas not yet formally published. To gain a more comprehensive understanding of the current research landscape, future studies could consider incorporating these additional avenues.

## Results

### Years of publication

**FIGURE 2:**
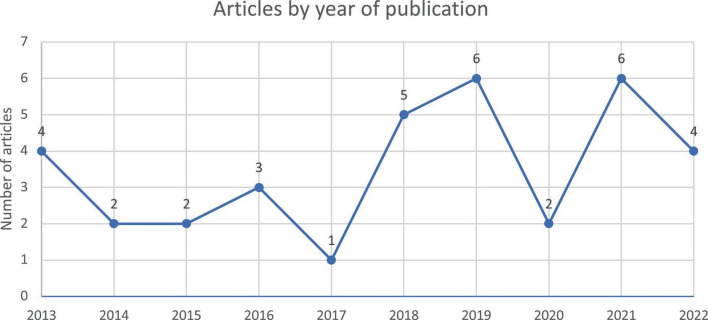
Number of articles retrieved by year of publication.

Figure 2 shows that the EMI policy in healthcare has started to gain the increased attention of researchers in the last five years, with the number of relevant studies increasing. Unfortunately, the number of studies published decreased in 2020, possibly due to the COVID-19 pandemic. These findings support other researchers’ views that EMI research in higher education is presently “trending” (2, 55). These findings also indicate that the effects of EMI policy have drawn the attention of healthcare education policy makers. However, research on EMI is in its infancy (56), and more research is needed to understand the EMI phenomenon.

### Locations of the studies

Table 2 presents the countries for which studies into EMI in healthcare education were conducted. Most EMI studies were undertaken in Saudi Arabia (7 out of 35), followed by South Africa (4) and India (3). From Table 2, we can infer that most of the studies were conducted in Asia and Africa, while there is a lack of EMI in healthcare education studies in South America countries, such as Brazil, Argentina, and Colombia. There is a need for collaborative research among researchers in different countries, which will return more generalizable findings and explore different country characteristics. Orduna-Nocito and Sánchez-García (3) stressed the need for more EMI research to determine patterns and trends emphasized by several researchers and international organizations. Table 2 supports UNESCO (4) recommendation that many developing countries should examine the language of instruction policy. Several Asian and African countries face EMI policy challenges that have not been examined by educational researchers and policymakers.

**TABLE 2:** Locations of the studies.

Region	Counts
Saudi Arabia	7
South Africa	4
India	3
Australia, Japan, Syria, and Thailand	2
Bahrain, Cambodia, China, Congo, Finland, Hong Kong, Jordan, Korea, and Ukraine	1
Combination of more than one country, a study in UK and China; a study in Nunavut and Greenland’ and a study in Netherlands, Saudi Arabia, United Kingdom, Egypt, United States, and Qatar. One study has no specified context	1

Studies in Australia, UK, and USA target non-English speaking students who study healthcare programs.

### Author’s affiliation and funding information

Using online tools, we determined whether there were any author Scopus ID numbers repeated more than once in the 35 articles. All of the researchers associated with these articles participated in publishing just one of the studies, except the following researchers who participated in two studies: Wilang, Jeffrey Dawala; Alshareef, Musab; Alrajhi, Ziyad; Alhamdan, Ali; Hamad, Bashir. This observation perhaps indicates that researchers are not continuing to instigate EMI policies in healthcare.

Funding: Ten studies were funded by the following organizations:

1. King Khalid University, Saudi Arabia
2. Taif University, Saudi Arabia
3. Japan Society for the Promotion of Science, Japan
4. King Abdulaziz University, Saudi Arabia
5. Asia Pacific Observatory of the World Health Organization, Cambodia
6. Suranaree University of Technology, Thailand
7. Norwegian Agency for Development Cooperation (Norad), Norway
8. King Abdullah International Medical Research Center, Saudi Arabia
9. Academy of Finland, Finland
10. Ministry of Education Humanities and Social Science Project, China

This list indicates that Saudi universities have the highest percentage of author affiliations (4). We found a relationship between the number of published studies and funding information. Interestingly, half of the published papers were funded by Saudi organizations, which shows that policymakers have gained an interest in EMI policy in a Saudi context. There is a need to fund projects in other countries such as African countries and South American countries. Supporting EMI research in other contexts will lead to more analyses of the phenomenon and more informative decisions by healthcare education policymakers.

### Journals and citations

The articles assessed here were published in several journals. The BMC Medical Education Journal published the highest number of these articles (4), followed by Asia-Pacific Education Researcher, Eastern Mediterranean Health, Health Professions Education, and Theory and Practice in Language Studies–each of these journals publishing two articles. The remaining journals published only one relevant article each (see online Supplementary materials).

In terms of citations in the Scopus database (databases such as Google Scholar have a different number of citations), the article by Joe and Lee (15) has the highest number of citations (64), followed by Dube and Mlotshwa (23) (19), Seabi et al. (16) (17), Alsuliman et al. (30) (13), Al Zumor (25) and Yang et al. (27) (12 each), and Mann et al. (45) (11). The remaining articles had fewer than 10 citations each. Eight articles had no citations.

### Sample of participants

Table 3 summarizes the findings of participant characteristics. Most often the participants were all students (in 20 articles), with the next most common participant cohort being students and instructors (8). Most of the studies involved only healthcare related majors (*n* = 32). Three studies involved participants from computer science, engineering, and social work in addition to healthcare participants.

**TABLE 3:** Description of the participants.

Level	Counts
Students	20
Students and teachers	8
Analysis of text	3
Educators and policymakers (decision makers)	2
Students, teachers, and administrators	2

### Instruments and methodological designs

Analysis of the research instruments that have been used in these studies shows that most of the researchers used solely a questionnaire (*n* = 10). The second most used tool is the test analysis, and the comprehension and analysis of policy documents, together (*n* = 5). Other studies used a questionnaires and interviews together (*n* = 3). Three studies used only interviews. Two studies used two questionnaires–one for students and another for instructors. The rest of the studies used one type of instrument or a combination of instruments, as we can see in Table 4.

**TABLE 4:** Other types of research instruments.

Level	Counts
Three questionnaires	1
Two questionnaires (one for teachers and one for students) and alumni records	1
Two questionnaires (one for teachers and one for students) and interviews	1
A survey, two discussion forums, and a workshop	1
A document and policy review, and key informant interviews with 16 agency representatives	1
Ethnographic observations, interviews, and audio-recorded interactions	1
A focus group and interviews	1
Interviews and questionnaires, observations, participant observations, reviews of news reports, government documents and report	1
Pre and posttest, and questionnaire	1
Questionnaire and focus group	1
Questionnaire and test	1
Two questionnaires and audiometry testing	1

**FIGURE 3:**
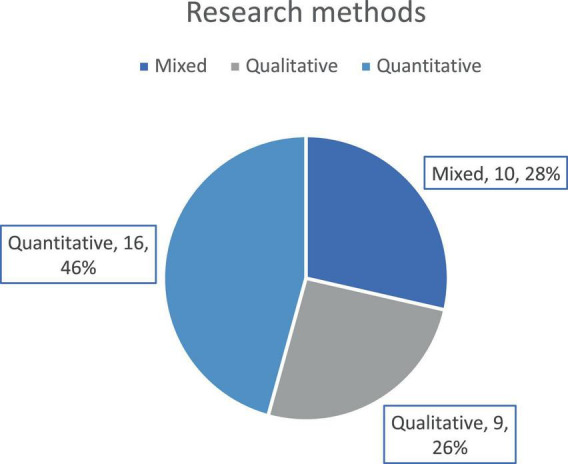
Preponderance of different research method designs.

Figure 3 shows that analysis procedures. Most of the studies used quantitative analysis (*n* = 16) followed by mixed methods analysis (*n* = 10). Only nine studies used qualitative analysis to analysis the findings.

## Main findings

The findings of 35 studies have revealed a spectrum of outcomes that reflect the complex interplay of factors influencing English medium instruction (EMI) in healthcare education. We will group them into five major themes.

### Theme 1: Learning and performance implications of EMI

**1.1. Negative impacts:** Al Zumor (25), Mann et al. (45), and Ndawo (39) present compelling evidence of the challenges posed by EMI. Al Zumor (25) reports that EMI adversely affects the comprehension of scientific content and overall student assessment, leading to negative emotions and suboptimal educational outcomes. Mann et al. (45) contribute to this narrative by demonstrating that medical undergraduates who learned English at a later age experience difficulties in speech discrimination amid background noise, a factor that is compounded by stress and is indicative of international students’ struggles. This contrasted with local students, who learned English earlier and exhibited better speech-noise ratio results.

**1.2. Neutral impact:** Joe and Lee (15) and Yang et al. (27) offer a different perspective by suggesting that EMI does not necessarily impede comprehension or academic success. Joe and Lee (15) note that students’ general English proficiency had no correlation with their understanding of lectures, indicating that other variables may be at play. Yang et al. (27) support this by highlighting the lack of significant differences in test scores between EMI and non-EMI students, although they identify four key challenges within EMI programs, including inadequate teaching materials and methods, which are mitigated by employing adaptive strategies such as using supplementary textbooks and enhancing self-learning skills, sometimes incorporating the Chinese language as a support tool.

**1.3. Positive outcomes:** Contrasting with the concerns about EMI, Alhamami and Almelhi (28) find that English fluency is a strong predictor of academic success, as evidenced by the higher cumulative GPA of English-speaking healthcare alumni in Saudi universities. Furthermore, Waterval et al. (41) observe that while the overall reception of EMI is positive, there are complexities in its practical application, especially for students who lack proficiency in the language of the patient population, complicating their workplace-based learning and interaction. These findings illustrate a complex landscape where EMI’s impact on healthcare education can vary widely. Factors such as the timing of English language acquisition, the proficiency of the learners, and the quality of instructional materials all play pivotal roles in determining the efficacy of EMI. It is clear from the diverse outcomes that a one-size-fits-all approach to EMI may not be feasible, and a nuanced understanding of the contextual factors at play is necessary to maximize the benefits of EMI while mitigating its potential drawbacks.

### Theme 2: Student and faculty attitudes toward EMI

**2.1 Positive attitudes:** Alfakhry et al. (22) and Horwood et al. (19) document a favorable disposition toward EMI among students and faculty, attributing this preference to the enhanced access to academic resources and potential for career progression that EMI offers. Alfakhry et al. (22) specifically found a consistent preference for EMI despite challenges stemming from insufficient Arabic medical translations. Horwood et al. (19) echoed these sentiments, noting that EMI provided significant opportunities for engagement with the wider scientific community and career development. However, they also pointed out the challenge of inadequate English competency among students, which could hinder the full realization of these benefits. Additionally, Pomat et al. (26) observed that both students and teachers acknowledge the necessity to improve their English skills to fully leverage the advantages of EMI.

**2.2. Negative attitudes and preference for mother tongue:** In contrast to the positive views, Al-Zubi et al. (20) and Alhamami and Almelhi (28) discovered a predilection for instruction in the mother tongue, driven by the barriers presented by EMI. Al-Zubi et al. (20) reported that while there was a general acceptance of Arabicized medical terms among students, the prevalent use of English for teaching and assessments, and the lack of comprehensive medical resources in Arabic, were significant obstacles. Alhamami and Almelhi (28) found a majority preference among students for receiving healthcare education in Arabic. Complementing these findings, Saha et al. (21) provided insights into the linguistic preferences of students from rural areas, with a substantial portion favoring Bengali, their mother tongue, as the medium of instruction. The studies collectively suggest that the debate on the efficacy of EMI versus mother tongue instruction in healthcare education remains unresolved, signaling a need for more context-specific research to determine the most effective language of instruction.

**2.3 Mixed attitudes:** Alshareef et al. (14) and Matthews and Van Wyk (17) present a more nuanced perspective, recognizing the dual nature of EMI’s impact. Alshareef et al. (14) found general support for EMI among decision-makers due to its global applicability, yet there was also an expressed interest in developing an Arabic curriculum for future use. Matthews and Van Wyk (17) observed an enhancement in communicative competence in students, but they also identified an ongoing need for additional linguistic support. Tayem et al. (57) reported varied responses related to the perceived language barrier in medical studies; while many students did not view language as an obstacle, there was a clear distinction between those proficient in English and those who were not. Interestingly, a significant majority of students were unsure of medical terms in Arabic, yet confident in their ability to communicate with patients in Arabic, revealing a dichotomy in language use and preference. The findings indicate a split in attitudes toward EMI, suggesting that while it has its proponents, there is considerable support for a bilingual approach that incorporates both English and Arabic. The analysis of attitudes toward EMI uncovers a complex interplay of factors, including resource availability, linguistic proficiency, and cultural considerations, that influence perceptions. These diverse perspectives highlight the importance of a tailored approach to EMI implementation that takes into account the unique linguistic and educational needs of healthcare students and professionals.

### Theme 3: Language proficiency and educational effectiveness

**3.1 Impact of language proficiency:** The critical link between language proficiency and educational effectiveness is elucidated in the research by Mann et al. (45) and Ndawo (39). Mann et al. (45) discovered significant disparities in speech-noise ratio performances among medical undergraduates based on the age at which they learned English. Those who acquired English later were disadvantaged in auditory processing in noisy environments, a challenge more pronounced among international students. This contrasted with local students who had learned English early and consequently had better auditory discrimination abilities.

Ndawo (39) extends this discussion by indicating that insufficient English proficiency undermines learner confidence, impedes the development of critical and reflective thinking, and complicates the comprehension of complex material. The study also notes that the effectiveness of nurse educators who are not proficient in EMI is significantly reduced, affecting the quality of instruction.

**3.2 Advantages of bilingual or hybrid instruction:** Alenezi and Kebble (58) and Alsuliman et al. (30) present a strong case for bilingual or hybrid instruction. Alenezi and Kebble (58) report that students showed a marked preference for code-switching, finding it more effective than a monolingual approach. Alsuliman et al. (30) reinforce this preference by showing that students performed better and responded faster when engaging with hybrid texts, which integrate both English and Arabic, compared to texts in only one language. Such bilingual strategies, as Mustonen and Strömmer (37) suggest, not only enhance comprehension of specific content but also allow students to utilize and develop their multilingual capabilities more strategically.

Yousif et al. (59) add to this narrative by demonstrating that a significant majority of students favored a combination of English and Arabic as the medium of instruction. Similarly, Kumar et al. (35) found a strong preference among students for instruction that incorporates both English and their native language, in this case, Hindi. These studies suggest that while EMI can present challenges, particularly for those with lower English proficiency, the integration of students’ first languages within the educational framework can lead to improved outcomes. The flexibility to switch between languages or to use a hybrid model can cater to the diverse needs of students, enhancing not only their understanding of the subject matter but also their overall academic performance.

### Theme 4: Factors influencing EMI effectiveness

**4.1 Instructor and student English proficiency:** The proficiency of instructors and students in English critically affects the effectiveness of EMI, as evidenced by Hijji (34) and Alrajhi et al. (13). Hijji (34) identified a lack of English language proficiency among healthcare instructors, manifesting in errors within exam questions and highlighting the necessity for adequate language skills among educators for reliable and valid assessments. Conversely, Alrajhi et al. (13) found that both students and faculty recognized the benefits of English proficiency, including improved access to medical information and enhanced job prospects, suggesting that a higher level of English competence can contribute positively to educational outcomes.

**4.2 Resource availability and support systems:** The accessibility of resources and support systems is paramount for the success of EMI, as indicated by Salamonson et al. (42) and Law et al. (29). Salamonson et al. (42) emphasized that factors such as age, enrollment status, and the primary language spoken at home can influence educational experiences in healthcare settings. Law et al. (29) noted the requirement for increased English proficiency among Cambodian medical professionals, advocating for more comprehensive English training to facilitate their participation in the ASEAN community. Dube and Mlotshwa (23) recognized that external factors, including parental involvement and technological resources, contribute to improved academic performance, while socioeconomic challenges and negative peer influences can be detrimental.

**4.3 Curriculum design and implementation challenges:** Roshini et al. (38), Khan (36), and Wilang and Nupong (60) discuss the complexities of curriculum design in the context of EMI. Roshini et al. (38) observed that dental students faced educational challenges when English was not the primary language of instruction, pointing to the need for curricula that accommodate diverse language backgrounds. Khan (36) argued for the inclusion of English for specific purposes (ESP) in higher education to address the needs of students who do not use English as a daily medium of communication. Wilang and Nupong (60) highlighted the variance in EMI experiences based on student and program characteristics, suggesting the need for curricular adjustments that consider English proficiency, student motivations, and the provision of additional language support. Overall, the effectiveness of EMI in healthcare education is influenced by a constellation of factors, encompassing language proficiency, resource allocation, and curriculum design. A nuanced approach that considers these variables is essential for the development of effective EMI strategies. Ensuring educators are well-versed in English, providing adequate resources and support, and designing curricula that address the specific linguistic and educational needs of the student body are critical steps toward optimizing the use of EMI in healthcare education.

### Theme 5: EMI policy and educational strategy

**5.1 Challenges and solutions in implementing EMI:** The implementation of EMI in higher education, particularly in healthcare and paramedical fields, comes with its own set of challenges and potential solutions, as discussed by Khan (36) and Alhamami and Almelhi (28). Khan (36) stresses the importance of a well-structured English curriculum tailored to the specific needs of learners who do not regularly use English outside the classroom. The study advocates for the integration of English for specific purposes (ESP) into the curriculum, suggesting that such an inclusion could significantly enhance the learning experience by aligning with the specific vocabulary and contexts students will encounter in their professional lives.

Alhamami and Almelhi (28) contribute to the discourse by analyzing alumni data, revealing that early grades in intensive English programs can be predictive of overall academic success. Their findings indicate that the perspectives of both students and instructors point to challenges in using EMI, particularly when students do not possess sufficient English fluency, resulting in potential hindrances to their academic achievement. This underscores the critical nature of English proficiency for both educators and learners and the need for robust support systems to facilitate effective EMI delivery.

Overall, these insights suggest that the success of EMI policies within healthcare education is contingent upon several interrelated factors. A concerted effort must be made to ensure that both students and instructors have the necessary proficiency in English. This includes providing access to resources such as specialized language courses and technological tools that support language learning and curriculum development. Additionally, the educational strategies employed must be thoughtfully designed to address and integrate the linguistic abilities of the learners to foster an environment where EMI can be a catalyst for educational advancement rather than a barrier.

## Recommendations

Based on the analysis of the selected studies, several strategies and solutions have been proposed to improve the quality of education in EMI policy healthcare programs.

Al Zumor (25) advocacy for “additive bilingual education” underscores the importance of solid English instruction in foundational years. This approach posits a tiered language program that begins with basic English education and methodically progresses to include specialized medical terminology. The challenge lies in implementing this without displacing the students’ native language, thus maintaining linguistic diversity while fostering English proficiency. A conducive environment for the use of Arabicized medical terms has been suggested by Al-Zubi et al. (20) to alleviate comprehension barriers. To operationalize this, the development of bilingual medical glossaries is necessary, requiring collaboration with native speakers in curriculum design and the rigorous vetting of such glossaries to ensure terminological accuracy across various Arabic dialects and contexts.

The reevaluation of language policies is another significant consideration, with Alenezi and Kebble (58) highlighting the pedagogical benefits of code-switching. The practical implementation of this would involve organizing training sessions for faculty to proficiently employ code-switching strategies in the classroom. Overcoming the potential resistance from teachers who are entrenched in traditional monolingual methods represents a significant hurdle. In areas where English is not the lingua franca, like Syria, the absence of professional medical translators in educational settings hampers learning. Al-Fakri et al. (22) have emphasized the urgent need to establish translation units within medical schools to support non-English-speaking students. This solution necessitates the recruitment and development of translators well-versed in medical terminology and language education, which may prove challenging given the required level of expertise.

Enhancing English proficiency across health programs has been stressed by Alhamami and Almelhi (28), pointing to the need for continuous English learning opportunities. The integration of English language modules tailored to healthcare into the curriculum would serve this purpose. However, the challenge arises in providing consistent and contextual language support to students throughout their education. The preference for EMI in medical education, as noted by Alrajhi et al. (13), suggests a future pursuit of Arabic for teaching medicine, alongside English, to enrich the learning environment. This dual-language approach would require the support of national educational and governmental bodies to overcome the significant challenge of aligning policy changes at a national level with the practical realities of medical education.

Alsuliman et al. (30) propose the use of simplified bilingual terminology to support learning among Arabic-speaking populations. This approach necessitates the development of educational materials that incorporate simplified terms in both languages, with the primary challenge being the maintenance of medical accuracy alongside linguistic simplification. The advocacy for the selection of well-qualified nursing students and the modernization of training facilities by Dube and Mlotshwa (23) points to the necessity for a rigorous student selection process and significant investments in infrastructure. However, securing the required funding and resources for such enhancements remains a daunting task. Concerns about the proficiency of instructors in test writing and item analysis raised by Hijji (34) have led to the recommendation for universities to offer workshops on these skills. The successful implementation of this recommendation hinges on the development of comprehensive faculty training programs and ensuring their participation and application of the training.

Horwood et al. (19) recognize EMI as a vehicle for overcoming language barriers in research partnerships, especially in low-income countries. They advocate for extensive support to develop English skills, aligning with the United Nations Sustainable Development Goals. The actionable step here involves forming partnerships with English training providers to offer language support for both staff and students, with the challenge being the integration of language training within the demanding schedule of research activities. Joe and Lee (15) have provided evidence supporting the efficacy of EMI in Korean higher education, asserting that EMI does not adversely affect student learning when the lectures are specialized and incorporate medical subjects, provided that the students’ general English proficiency is adequate. Kumar et al. (35) noted that the educational needs of Indian students differ from Western students, necessitating tailored didactic lectures to enhance comprehension. This suggests that traditional teaching methods, such as using chalkboards, may be more effective than modern technology like PowerPoint presentations in certain contexts. Lazer-Pankiv and Pysmenna (31) suggested the development and implementation of uniform standards for phonetic and orthographic adaptations of medical terminology in English. This comprehensive approach would require the preservation of etymological principles and the careful selection of terminology to maintain medical precision.

Matthews and Van Wyk (17) shed light on the disconnect between language learning and practical communication skills. Their study of learners in an isiZulu language program demonstrated improved language knowledge and attitudes but did not extend to effective patient communication. This gap underscores the necessity for communicative language teaching methods to be honed, ensuring that learners are not only proficient in the language but are also capable of practical communication with patients, which is crucial in healthcare settings. Møller (40) draws attention to the critical demand for Inuit nurses who are versed in the Arctic health system. The lived experiences and insights of Inuit nurses are invaluable, he argues, in shaping education and health systems that are responsive to the unique needs of Arctic communities. Here, the support for and retention of nursing students and practitioners in the Arctic become imperative, demanding both educational and systemic interventions that acknowledge and build upon the distinct knowledge and skills pertinent to the region. Pun (18) offers a perspective on the educational bridge between veterinary studies and clinical practice in bilingual contexts. He posits that the development of multimodal teaching and learning materials that are culturally contextual can address the communication challenges that arise from language discrepancies in such settings. This approach recognizes the necessity of a pedagogical strategy that is flexible and responsive to the linguistic and cultural nuances of veterinary education.

Roshini et al. (38) emphasized the integration of language skills, communication abilities, and behavioral sciences in dental education. They argue that the incorporation of these elements is fundamental to the smooth transition from school to dental college, enabling students to not only excel academically but also to effectively serve their patients upon entering the professional field. Seabi et al. (16) offered a counter-narrative to the perceived linguistic privilege of native English speakers in multilingual educational contexts. They observe that Caucasian students in South Africa, lacking proficiency in indigenous African languages, experienced limitations in their ability to serve clients effectively. This finding challenges the assumption that native English speakers inherently hold an advantage in multilingual settings, suggesting that a multilingual proficiency is an asset in the diverse linguistic landscape of professional healthcare programs. Yang et al. (27) highlight the complexities inherent in initiating EMI programs, which necessitate extensive faculty development, organizational backing, and the implementation of successful learning strategies for students and groups. They note that adaptive strategies deployed by both educators and students can serve as invaluable blueprints for enhancing the efficacy of EMI programs across diverse educational landscapes.

Yousif et al. (59) provide pragmatic suggestions aimed at augmenting the teaching methods for Saudi pharmacy students. They advocate for the adoption of interactive teaching methodologies and the employment of bilingual educational media to reinforce knowledge transfer and elicit active student engagement. Such approaches have the potential to not only enrich the learning experience but also to ensure a deeper comprehension of the subject matter. Waterval et al. (41) document the unique academic opportunities that medical curriculum partnerships offer to students. They argue for the adaptation of home curricula to the health systems of host countries, a strategy that not only provides students with a comprehensive international healthcare perspective but also maintains the relevance and accreditation of the curriculum globally. These recommendations represent a composite vision for advancing healthcare education through EMI, each with actionable steps and challenges that must be navigated. Successful application of these recommendations would involve not only strategic planning and resource allocation but also an openness to pedagogical innovation and cultural sensitivity.

## Conclusion

English as a medium of instruction research in healthcare education has gained more attention in the last five years, but the published studies do not provide sufficient evidence about its impact. The language of instruction was not the main topic of the analyzed studies, but was instead discussed as a sub-topic or a marginalized factor within other main factors. Future research should focus on the language of instruction in healthcare education. Most of the authors of the present study are not language specialists and do not have a background in applied linguistics topics. There is a need for collaboration between language specialists and healthcare educationalists in order to conduct more thorough research about EMI in healthcare. Most of the studies we examined were conducted in Arabic speaking countries. There is a need to explore South American and African contexts. Future studies should collaborate between researchers from different countries to provide more comprehensive outcomes of current EMI policy. The same authors of the studies assessed in the present report always published just one of those studies. This might show a lack of support from institutions in terms of investigating EMI within healthcare education. Healthcare education institutions should provide more funds for research into studies about EMI in healthcare education. EMI is an interdisciplinary topic that interests healthcare journals and applied linguistics journals. EMI research was published in both healthcare journals and language journals. We recommend having a special issue about EMI within the context of healthcare in one of these journals. The main repeated topic in the analyzed studies was stakeholders’ perceptions and attitudes. Most of the studies focus on students as participants, and attitude as a topic. There is a need for more experimental studies and empirical studies that examine the influence of EMI on students’ achievements in healthcare education. Future studies should also include different stakeholders’ views in one investigation. Most of the studies are quantitative and used closed-ended questionnaires. There is a need for mixed methods research. Future studies should adapt rigorous components of a mixed methods research design to achieve data integration. The findings of the current studies show conflicted results, and the use of well-designed studies will resolve several concerns about learning processes within the EMI context.

## Data availability statement

The original contributions presented in this study are included in this article/Supplementary material, further inquiries can be directed to the corresponding author.

## Author contributions

MA: Writing – original draft, Writing – review & editing.
